# Genetic Characterization of Novel H7Nx Low Pathogenic Avian Influenza Viruses from Wild Birds in South Korea during the Winter of 2020–2021

**DOI:** 10.3390/v13112274

**Published:** 2021-11-13

**Authors:** Yu-Na Lee, Dong-Hun Lee, Jung-Hoon Kwon, Jae-In Shin, Seo Yun Hong, Ra Mi Cha, Yoon-Gi Baek, Eun-Kyoung Lee, Mingeun Sagong, Gyeong-Beum Heo, Kwang-Nyeong Lee, Youn-Jeong Lee

**Affiliations:** 1 Avian Influenza Research & Diagnostic Division, Animal and Plant Quarantine Agency, 177 Hyeoksin 8-ro, Gimcheon-si 39660, Gyeongsangbuk-do, Korea; sji6951@naver.com (J.-I.S.); yuuunnn0116@gmail.com (S.Y.H.); rami.cha01@korea.kr (R.M.C.); bygttaggg@gmail.com (Y.-G.B.); ensenble@korea.kr (E.-K.L.); sagongmg@korea.kr (M.S.); imheo@korea.kr (G.-B.H.); leekwn@korea.kr (K.-N.L.); leeyj700@korea.kr (Y.-J.L.); 2 Department of Pathobiology and Veterinary Science, The University of Connecticut, 61 North Eagleville Road, Unit-3089, Storrs, CT 06269, USA; dong-hun.lee@uconn.edu; 3 College of Veterinary Medicine, Kyungpook National University, 80, Daehak-ro, Buk-gu, Daegu 41566, Korea; junghoon.kwon@knu.ac.kr

**Keywords:** avian influenza virus, H7, reassortant, phylogenetic analysis, wild bird

## Abstract

Zoonotic infection with avian influenza viruses (AIVs) of subtype H7, such as H7N9 and H7N4, has raised concerns worldwide. During the winter of 2020–2021, five novel H7 low pathogenic AIVs (LPAIVs) containing different neuraminidase (NA) subtypes, including two H7N3, an H7N8, and two H7N9, were detected in wild bird feces in South Korea. Complete genome sequencing and phylogenetic analysis showed that the novel H7Nx AIVs were reassortants containing two gene segments (hemagglutinin (HA) and matrix) that were related to the zoonotic Jiangsu–Cambodian H7 viruses causing zoonotic infection and six gene segments originating from LPAIVs circulating in migratory birds in Eurasia. A genomic constellation analysis demonstrated that all H7 isolates contained a mix of gene segments from different viruses, indicating that multiple reassortment occurred. The well-known mammalian adaptive substitution (E627K and D701N) in PB2 was not detected in any of these isolates. The detection of multiple reassortant H7Nx AIVs in wild birds highlights the need for intensive surveillance in both wild birds and poultry in Eurasia.

## 1. Introduction

Avian influenza virus (AIV) subtype A (H7) has raised global concerns because it has been a leading cause of zoonotic infections over the past two decades [1]. In particular, Asian lineage low pathogenic (LP) and highly pathogenic (HP) H7N9 viruses have posed a significant challenge to both public health and the poultry industry since their emergence in 2013 and 2017, respectively [2,3]. The H7N9 virus has caused five waves of virulent human infections in China, resulting in significant economic loss [4]. Although the prevalence of the H7N9 virus in poultry has decreased dramatically since the application of the H7N9 poultry vaccine in China, the H7N9 viruses have not been eradicated from poultry and are still detected in live-bird markets (LBMs) in China [5,6].

In early 2018, a severe H7N4 infection was confirmed in a 68-year-old woman in Jiangsu, China, which was the first human case of infection with H7N4 LPAIV [7]. Genetically similar H7N4 viruses were subsequently detected in the patient’s backyard poultry, substantiating the belief that the infection was zoonotic. Phylogenetic analysis revealed that the H7N4 viruses were genetically distinct from the zoonotic H7N9 viruses in China [8]. In addition, novel H7N4, H7N5, and H7N6 reassortant LPAIVs possessing the hemagglutinin (HA) gene derived from the zoonotic H7N4 virus have been identified in ducks from LBMs and slaughterhouses in Cambodia since February 2018 [9,10]. Phylogenetic analysis showed that the Jiangsu–Cambodian H7 HA genes emerged during late 2017 and were derived from H7N7 and H7N2 viruses previously detected in aquatic birds in East Asia [9].

In South Korea, nationwide AIV surveillance has been conducted to rapidly detect and respond to potential introductions and outbreaks of H5 and H7 LPAI and HPAI [11]. During the winter of 2020–2021, we isolated five H7 LPAIVs sharing recent common ancestry with the Jiangsu–Cambodian H7 HA gene from wild birds in South Korea. To better understand the evolutionary history, genetic diversity, and zoonotic potential of these H7 LPAIVs, we sequenced the full-length genomes of the isolates and analyzed their genetic characteristics.

## 2. Materials and Methods

A total of 16,293 samples (fresh fecal samples, oropharyngeal or cloacal swabs, and carcasses) were collected from major migratory habitats in South Korea between October 2020 and April 2021. Sample collection was conducted by the Livestock Health Control Association or by regional veterinary offices according to the national surveillance program in South Korea. Samples were examined by virus isolation in 9–11-day-old specific-pathogen-free (SPF) embryonated eggs. After incubation for 4 days at 37 °C, the eggs were chilled, and the allantoic fluid was harvested and determined by hemagglutination assay using chicken erythrocytes. Host-species identification was confirmed using a barcoding system utilizing mitochondrial DNA from feces, as previously described [12].

The whole genome was amplified, and next-generation sequencing was conducted using the Illumina MiSeq platform, as described previously [13]. Trimmed and filtered NGS reads were assembled de novo using CLC genomics workbench software. The genome sequences of the viruses isolated in this study were deposited in the GISAID EpiFlu database under accession numbers EPI_ISL_3663323–EPI_ISL_3663327. All available sequence information of AIVs was retrieved in April 2021 for isolates collected in Asia and North America, using the GenBank (https://www.ncbi.nlm.nih.gov/genomes/FLU, (accessed on 30 April 2021)) and GISAID EpiFlu (http://www.gisaid.org, (accessed on 30 April 2021)) databases, and used for comparative phylogenetic analysis.

The maximum likelihood (ML) phylogenetic trees for all genes were constructed with RAxML version 8.2.12 using a gamma distribution and a general time-reversible model with 1000 rapid bootstrap replicates [14]. The genotype was analyzed according to the tree topology and a nucleotide sequence identity of >97%, regarded as significant when the bootstrap support value was >90. To estimate the time of the most recent common ancestry (tMRCA), Bayesian relaxed clock phylogenetic analysis of the HA gene was performed using the BEAST v1.10.4 package [15]. The Bayesian phylogenetic tree was inferred using the HKY + G nucleotide substitution model [16]. The Gaussian Markov random field (GMRF) Bayesian skyride coalescent tree prior and uncorrelated relaxed (UCLD) clock models were selected for a flexible approach [17]. The Markov chain Monte Carlo (MCMC) analysis was run in parallel for two chains, each with 50 million steps, with the parameters and trees sampled every 5000 steps. The resulting log and tree files were combined using LogCombiner v1.10.4 (https://beast.community/logcombiner (accessed on 30 April 2021)) after a 10 percent burn-in, yielding a total of 18,002 parameters and posterior trees. The parameters were analyzed with Tracer v1.7.2 (http://tree.bio.ed.ac.uk/software/tracer/ (accessed on 30 April 2021)), and all parameters had an effective sample size greater than 200. A maximum clade credibility (MCC) tree was generated using TreeAnnotator in BEAST and visualized using FigTree 1.4.2 (http://tree.bio.ed.ac.uk/software/figtree/ (accessed on 30 April 2021)).

## 3. Results and Discussion

Between October 2020 and April 2021, a total of 291 AIVs were isolated from wild bird habitats in South Korea. The proportion of H7 viruses isolated was 5.8% (17/291). In addition, no LPAIVs, except for subtype H9N2 viruses, were detected in commercial poultry under nationwide surveillance in the same period. In a preliminary phylogenetic analysis of the HA gene, we identified five H7 AIVs genetically similar to the Jiangsu–Cambodian H7 gene. The five H7 AIVs contained different neuraminidase (NA) subtypes, including an H7N8 and an H7N9 detected from Jeollabuk-do Province in November 2020, and two H7N3 and an H7N9 from Jeju Province and Chungcheongbuk-do Province from January to March 2021 (Table 1). The host species of the H7 isolates identified using the DNA barcoding technique were mallard or spot-billed duck, which are dominant dabbling duck species in autumn and winter in South Korea [18]. Based on previous studies, it should be noted that the DNA barcoding technique has a limitation in classifying some sister species within genera, including mallard and spot-billed duck [19,20]. Consistent with the previous finding, we were not able to distinguish whether the host species of four isolates was mallard or spot-billed duck. Therefore, the host name of undistinguishable fecal samples was designated as wild duck.

**Figure 1:**
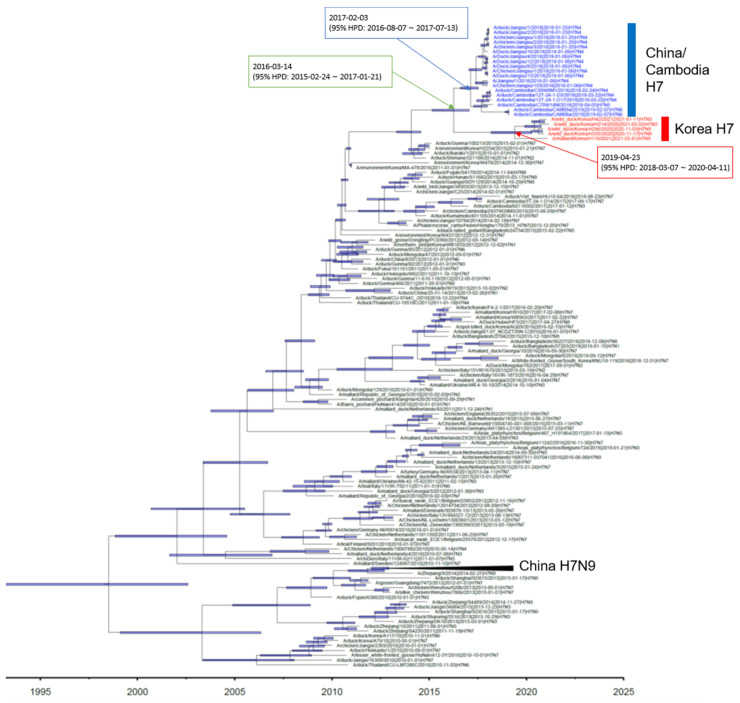
Maximum clade credibility phylogeny of the HA gene sequences of H7Nx avian influenza viruses in Eurasia. We estimated the maximum clade credibility tree using a time-measured Bayesian phylogenetic model implemented in BEAST v1.10.4. The Hasegawa–Kishino–Yano nucleotide substitution model, with a discrete gamma distribution with four categories, was applied. Branch lengths represent time. Horizontal bars indicate 95% Bayesian credible intervals for estimates of common ancestry. The H7Nx viruses isolated in China and Cambodia are colored with blue. The H7Nx viruses isolated in this study are colored with red. The estimated time of the most recent common ancestry with 95% highest posterior density (HPD) is displayed on the node of cluster.

Phylogenetic analysis showed that the HA gene of the H7 isolates from wild ducks in South Korea formed a monophyletic cluster, and the tMRCA was estimated to be 24 April 2019 (95% highest posterior density (HPD): from 16 March 2018–17 April 2020) (Figure 1 and Supplementary Figure S1). It shared recent common ancestry with the Jiangsu–Cambodian H7 cluster, which consists of the H7Nx viruses detected in Jiangsu, China, and Cambodia. The estimated tMRCA between the Jiangsu–Cambodian H7 cluster and the five H7 isolates derived from wild ducks in South Korea was estimated to be 2 March 2016 (95% HPD: 18 February 2015–25 January 2017). The tMRCA of the Jiangsu–Cambodian H7 viruses was estimated to be 29 January 2017 (95% HPD: 26 July 2016–24 July 2017). The long branch length in the HA phylogeny between the Korean H7 cluster and the Jiangsu–Cambodian H7 cluster, together with the relatively low HA gene nucleotide sequence identity (97.03–97.74%) and the estimated tMRCA, suggest that the ancestral viruses had been circulating undetected. However, the details of the evolutionary history of these viruses remain uncertain due to the lack of surveillance data.

The phylogenies of NA genes indicated that all subtypes of the NA gene (N3, N8, and N9) of the isolates belonged to the Eurasian lineage (Supplementary Figure S2). The N9 gene of two H7N9 isolates was phylogenetically distinct from that of the zoonotic H7N9 viruses in China (Supplementary Figure S2) and shared the highest nucleotide sequence identity (>98.6%) with that of a wild-bird-origin LPAIV, A/mallard/Kagoshima/KU-KGS6/2018 (H11N9) (Table 2).

**Figure 2:**
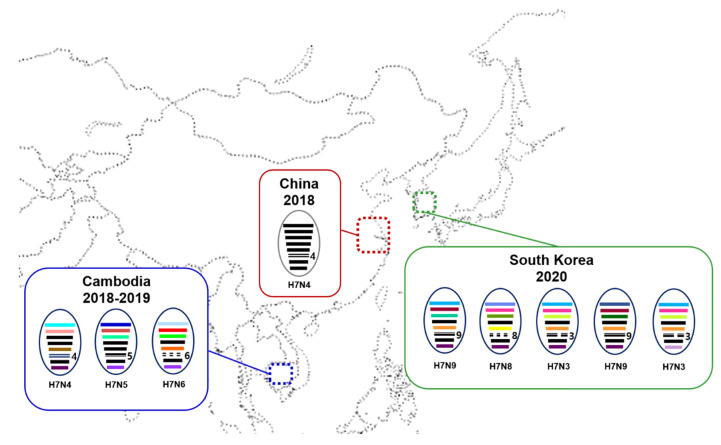
Schematic diagram of genomic compositions of the Jiangsu–Cambodian H7Nx viruses in East Asia. Each oval represents a viral isolate, within which the eight gene segments (from top to bottom: PB2, PB1, PA, HA, NP, NA, M, and NS) are indicated by horizontal bars. The color of each bar identifies its origin. Jiangsu province in China is indicated by red dotted box. The Cambodia and South Korea are indicated by blue and green dotted box, respectively.

A genomic constellation analysis demonstrated that all H7 isolates from South Korea contained a mix of gene segments from different viruses (Figure 2), suggesting that the H7 viruses seem to have acquired their gene segments from different gene pools of Eurasian LPAIVs through reassortment. Two H7N9 viruses had different genotypes, each of which had different PB2 and PA segments. On the other hand, two H7N3 viruses isolated from Jeju Island in January and March 2021 differed only in the NS segment. It has also been reported that none of the Cambodian H7Nx viruses shared all segments with the Jiangsu H7N4 viruses, indicating that these viruses also obtained gene segments through reassortment [9,10]. The H7Nx isolates in Korea had HA and M genes that were genetically similar to the Jiangsu–Cambodian H7 viruses, and six genes that most likely originated from LPAIVs circulating in migratory birds in Eurasia. It has been shown that AIVs in migratory aquatic birds generally continually form diverse and transient gene constellations through reassortment [21]. We assume that the H7 reassortant viruses were produced within the East Asian–Australasian (EAA) flyway, since Cambodia, China, and Korea are situated within the EAA flyway. The diverse gene constellations and tMRCAs of the viruses suggest that these viruses have been circulating in the wild bird population in East Asia for at least 1 year prior to the first detection of the virus in humans and poultry in 2018 and have evolved through frequent reassortment.

The amino acid sequence of the HA protein of all H7 isolates in this study contained a monobasic arginine residue at the cleavage site (PELPKGR/GLF), indicating a low-pathogenicity phenotype in chickens [22] (Table 3). The amino acids at positions 226 and 228 (H3 numbering) of the receptor-binding sites of the HA1 proteins of the H7 isolates indicated a preference for α2,3-linked sialic acid receptors rather than α2,6-linked sialic acid receptors [23]. Consistent with previous findings for the Jiangsu–Cambodian H7 viruses isolated from poultry, well-known mammalian adaptive markers [24], such as E627K and D701N in PB2, were not detected in any of the H7 isolates from South Korea.

Recently reported human infection with the H7N4 LPAIV has raised concerns about the pandemic potential of the virus, even though there was no evidence that human-to-human transmission has occurred. Considering that the pathogenicity in mice of the H7N6 virus belonging to the Jiangsu–Cambodian cluster was similar to that of the Chinese H7N9 viruses [10], the risk of interspecies transmission of the viruses should not be underestimated. Therefore, the recent detection of H7 viruses similar to the Jiangsu–Cambodian H7Nx viruses in wild birds emphasizes the need for continued surveillance in both wild birds and poultry in Eurasia; this will provide early warning of novel H7Nx virus threats to poultry as well as the potential risk to public health.

## Figures and Tables

**Table 1:** H7 isolates used in this study (*n* = 5).

Virus Name	Subtype	Samples	Collection Date	Region	Latitude/Longitude	GISAID Isolate ID
A/wDK/Kr/H296/2020	H7N9	Feces	2020-11-03	JB	35°46′4.9692″ N/126°38′22.6278″ E	EPI_ISL_3663323
A/wDK/Kr/H333/2020	H7N8	Feces	2020-11-17	JB	35°47′56″ N/126°46′16″ E	EPI_ISL_3663324
A/wDK/Kr/H42/2021	H7N3	Feces	2021-01-11	JJ	33°30′19.22″ N/126°53′31.18″ E	EPI_ISL_3663325
A/mallard/Kr/H118/2021	H7N9	Feces	2021-02-01	CB	36°39′9.5004″ N/127°22′40.2096″ E	EPI_ISL_3663326
A/wDK/Kr/H214/2021	H7N3	Feces	2021-03-02	JJ	33°30′22.27″ N/126°53′33.53″ E	EPI_ISL_3663327

GISAID: Global Initiative on Sharing All Influenza Data; wDK: wild duck; Kr: Korea; JB: Jeollabuk-do; JJ: Jeju-do; CB: Chungcheongbuk-do.

**Table 2:** Nucleotide identity of near homologs in GenBank and GISAID related to the isolates.

Gene	Virus with the Highest Nucleotide Sequence Identity [% Nucleotide Sequence Identity]				
	H296/20 (H7N9)	H333/20 (H7N8)	H42/21 (H7N3)	H118/21 (H7N9)	H214/21 (H7N3)
PB2	A/Anas platyrhynchos/South Korea/JB29-91-95/2019 (H11N2) [99.61%]	A/barnacle goose/Netherlands/2/ 2014 (H3N6) [98.07%]	A/duck/Bangladesh/ 41797/2019 (H3N8) [98.90%]	A/duck/Kumamoto/ 431107/2014 (H1N4) [99.04%]	A/duck/Bangladesh/ 41797/2019 (H3N8) [98.90%]
PB1	A/mallard/Anhui/3-683/ 2019 (H6N2) [99.47%]	A/Anas platyrhynchos/South Korea/JB31-96/2019 (H11N9) [98.94%]	A/mallard/Anhui/3-617/ 2019 (H6N1) [98.72%]	A/mallard/Anhui/3-683/ 2019 (H6N2) [98.94%]	A/mallard/Anhui/3-617/2019 (H6N1) [98.72%]
PA	A/mallard/South Korea/KNU2019-54/2019 (H5N3) [99.49%]	A/duck/Mongolia/543/ 2015 (H4N6) [99.63%]	A/duck/Guangdong/ H31/2020 (H3N8) [99.07%]	A/duck/Mongolia/398/ 2018 (H3N8) [98.79%]	A/duck/Guangdong/ H31/2020 (H3N8) [99.11%]
HA	A/Jiangsu/1/2018(H7N4) [97.74%]	A/Jiangsu/1/2018 (H7N4) [97.68%]	A/Jiangsu/1/2018(H7N4) [97.62%]	A/Jiangsu/1/2018(H7N4) [97.03%]	A/Jiangsu/1/2018 (H7N4) [97.50%]
NP	A/mallard/Anhui/2-549/ 2019 (H6N1) [99.33%]	A/mallard/South Korea/KNU2019-51/2019 (H5N3) [99.60%]	A/teal/Egypt/ MB-D-487OP/2016 (H7N3) [99.06%]	A/teal/Egypt/ MB-D-487OP/2016 (H7N3) [99.0%]	A/teal/Egypt/ MB-D-487OP/2016 (H7N3) [99.13%]
NA	A/mallard/Kagoshima/ KU-KGS6/2018 (H11N9) [98.73%]	A/mallard/China/T222(3)/2018 (H3N8) [98.73%]	A/mallard/South Korea/JB21-58/2019 (H5N3) [99.29%]	A/mallard/Kagoshima/ KU-KGS6/2018 (H11N9) [98.58%]	A/mallard/South Korea/JB21-58/2019 (H5N3) [99.36%]
M	A/duck/Mongolia/ MN18-1/2018 (H3N6) [100%]	A/duck/Mongolia/ MN18-1/2018 (H3N6) [99.59%]	A/duck/Mongolia/ MN18-1/2018 (H3N6) [99.69%]	A/duck/Mongolia/ MN18-1/2018 (H3N6) [99.69%]	A/duck/Mongolia/ MN18-1/2018 (H3N6) [99.39%]
NS	A/green-winged teal/South Korea/KNU2019-72/2019 (H3N8) [99.40%]	A/green-winged teal/South Korea/KNU2019-72/2019 (H3N8) [99.40%]	A/green-winged teal/South Korea/KNU2019-72/2019 (H3N8) [99. 28%]	A/green-winged teal/South Korea/KNU2019-72/2019 (H3N8) [98. 80%]	A/Environment/Jiangxi/12590/2019 (H10N3) [99.40%]

PB: polymerase basic protein; PA: polymerase acidic protein; HA: hemagglutinin; NP: nucleocapsid protein; NA: neuraminidase; M: matrix protein; NS: nonstructural protein. H296/20: A/wild duck/Kr/H296/2020; H333/20: A/wild duck/Kr/H333/2020; H42/21: A/wild duck/Kr/H42/2021; H118/21: A/mallard/Kr/H118/2021; H214/21: A/wild duck/Kr/H214/2021.

**Table 3:** Molecular characteristics associated with viral pathogenicity of H7 isolates.

Virus ^†^	Subtype	HA ^‡^			PB2		PB1	PA	NA			M2
		Cleavage Site	226	228	627	701	368	409	Stalk del.	274	292	31
A/wDK/Kr/H296/2020	H7N9	PELPKGR/GLF	Q	G	E	D	I	S	No	H	R	S
A/wDK/Kr/H333/2020	H7N8	PELPKGR/GLF	Q	G	E	D	I	S	No	H	R	S
A/wDK/Kr/H42/2021	H7N3	PELPKGR/GLF	Q	G	E	D	I	S	No	H	R	S
A/mallard/Kr/H118/2021	H7N9	PELPKGR/GLF	Q	G	E	D	I	S	No	H	R	S
A/wDK/Kr/H214/2021	H7N3	PELPKRR/GLF	Q	G	E	D	I	S	No	H	R	S
A/DK/Cambodia/CAM08a/2019	H7N6	PELPKGR/GLF	Q	G	E	D	I	S	No	H	R	S
A/DK/Cambodia/CAM09a/2019	H7N6	PELPKGR/GLF	Q	G	E	D	I	S	No	H	R	S
A/Jiangsu/1/2018	H7N4	PELPKGR/GLF	Q	G	K	D	I	S	No	H	R	S
A/CK/Jiangsu/1/2018	H7N4	PELPKGR/GLF	Q	G	E	D	I	S	No	H	R	S
A/DK/Jiangsu/4/2018	H7N4	PELPKGR/GLF	Q	G	E	D	I	S	No	H	R	S
A/DK/Cambodia/12T-24-1-D3/2018	H7N4	PELPKGR/GLF	Q	G	E	D	I	S	No	H	R	S
A/DK/Cambodia/C50W8M1/2018	H7N4	PELPKGR/GLF	Q	G	E	D	I	S	No	H	R	S
A/Anhui/1/2013	H7N9	PEIPKGR/GLF	L	G	E	D	V	N	Yes	H	R	N
A/Shanghai/1/2013	H7N9	PEIPKGR/GLF	Q	G	E	D	I	N	Yes	H	R/K	N

^†^ wDK: wild duck; DK: duck; CK: chicken. ^‡^ H3 numbering was used. HA: hemagglutinin; PB: polymerase basic; PA: polymerase acidic; NA: neuraminidase; M: matrix.

## Data Availability

Data are contained within the article; no additional data have been used.

## Supplementary Materials

The following are available online at www.mdpi.com/article/10.3390/v13112274/s1. Supplementary Figure S1. The maximum likelihood phylogenetic tree for the H7 gene. Supplementary Figure S2. The maximum likelihood phylogenetic tree for the PB2 (A), PB1 (B), PA (C), NP (D), N3 (E), N8 (F), N9 (G), MP (H), and NS (I) genes of five H7 viruses isolated in South Korea, along with complete sequences of genes of avian influenza viruses available in GISAID and the NCBI Influenza Virus database. Evolutionary analyses were conducted in RAxML. Clusters were assigned on the basis of tree topology and a nucleotide sequence identity of >97%, regarded as significant when the bootstrap support value was >90. Supplementary Table. Acknowledgement table from GISAID for samples included in the paper.
